# The complete chloroplast genome sequence of the *Cucurbita pepo* L. (Cucurbitaceae)

**DOI:** 10.1080/23802359.2018.1483766

**Published:** 2018-06-29

**Authors:** Chen Zhang, Qianglong Zhu, Shi Liu, Peng Gao, Zicheng Zhu, Xuezheng Wang, Feishi Luan

**Affiliations:** a College of Horticulture and Landscape Architecture, Northeast Agricultural University, Harbin, Heilongjiang, China;; b Key Laboratory of Biology and Genetic Improvement of Horticulture Crops (Northeast Region) Ministry of Agriculture, Harbin, Heilongjiang, China

**Keywords:** *Cucurbita pepo* L., chloroplast genome, summer squash, zucchini

## Abstract

*Cucurbita pepo* is an important economic plant cultivated widely in the world. The complete chloroplast genome sequence of *C. pepo* is reported here. The genome is 157,343 bp in length and exhibits a typical quadripartite structure of the large (LSC, 87,970 bp) and small (SSC, 18,167 bp) single-copy regions, separated by a pair of inverted repeats (IRs, 25,603 bp). A total of 131 genes were predicted including 85 protein-coding genes, eight rRNA genes and 38 tRNA genes. Further, phylogenetic analysis showed that *C. pepo* were closely related to other species in the family Cucurbitaceae. The complete chloroplast genome of *C. pepo* would be taken as a useful molecular tool for species discrimination, taxonomy, and phylogenetic relationships in the family Cucurbitaceae.

*Cucurbita pepo* L. is a biologically and economically important crop (Montero-Pau et al. 2017), commonly known as ‘Zucchini’ or ‘Summer squash’. The species with high genetic diversity is a world-wide cultivated vegetable of American origin (Formisano et al. 2012). Chloroplast genome is maternally inherited in *C. pepo*, and considered a useful molecular tool to discriminate species, explore species divergence, and trace biogeographic history (Lim et al. 1990; Schaefer et al. 2009; Logan et al. 2015). Moreover, the pericarp pigment would be regulated by the different expression of genes in the plastid genome to show different skin color (Schaffer et al. 1984) and the cotyledon senescence has been delayed by the expression of plastid-encoded genes (Mishev et al. 2011). In this study, we characterized the complete chloroplast genome sequence of *Cucurbita pepo* for promoting the studies on the phylogenetic relationships, germplasm exploration, genetic breeding, and physiological mechanism.

The genomic DNA was isolated from the leaves of *C. pepo* subsp. *pepo* (Accession: BGV004370) using the CTAB method as previously described (Montero-Pau et al. 2017). Genomic DNA was subjected to construct a 500 bp pair-end library and sequenced by illumina HiSeq2000 (Macrogen, Seoul, Republic of Korea). The whole genome sequence data have been deposited for academic use in the SRA database of NCBI with the accession (SRR5581782), it was downloaded and trimmed, high quality pair-end reads (2 × 101 bp) of 0.23 Gb (∼0.8× coverage to nuclear genome) were randomly extracted using Seqtk and assembled with using the Plasmidspades.py in SPAdes (v3.10.1) (Bankevich et al. 2012). Contigs representing the chloroplast genome were retrieved, ordered and joined into a single draft sequence by comparison with the chloroplast genome of *Cucumis melo* (GenBank accession no. NC_015983.1) as a reference (Rodriguez-Moreno et al. 2011). The gaps in the chloroplast single draft sequence of were closed by using GapCloser (v1.12-r6). The draft sequence was then confirmed and manually corrected by PE read mapping. Finally, the draft sequence was annotated using the two integrated web servers, CpGAVAS (Chang et al. 2012) and DOGMA (Wyman et al. 2004), and manually corrected by visual inspection using IGV (Robinson et al. 2011).

A typical chloroplast genome consists of four distinct regions, a large and a small single copy region (LSC and SSC, respectively) separated by two inverted repeat regions (IRa and IRb). The complete chloroplast genome of *Cucurbita pepo* (GenBank accession number MH031787) is 157,343 bp in length with 37.16% GC contents, and exhibits a typical quadripartite structure, consisting of a pair of IRs (25,603 bp) separated by the LSC (87,970 bp) and SSC (18,167 bp) regions. There is a total of 131 genes, including 85 protein-coding genes, eight rRNA genes and 38 tRNA genes; six of the protein-coding genes, six of the tRNA genes and four rRNA genes are duplicated within the IRs.

To determine the phylogenetic position of *C. pepo*, a phylogenetic analysis was carried out among 17 complete chloroplast genomes that are derived from Cucurbitaceae, Leguminosae, Cruciferae, Solanaceae, and Gramineae. The phylogenetic tree was constructed by maximum-likelihood method using the program PhyML in Unipro UGENE v1.29.0 (Okonechnikov et al. 2012). The results showed that *C. pepo* was clustered into the family Cucurbitaceae and closer to Gynostemma than Citrullus, Coccinia, and Cucumis (Figure 1). In addition, other species were also well clustered into the clades corresponding to their families.

**Figure 1.**
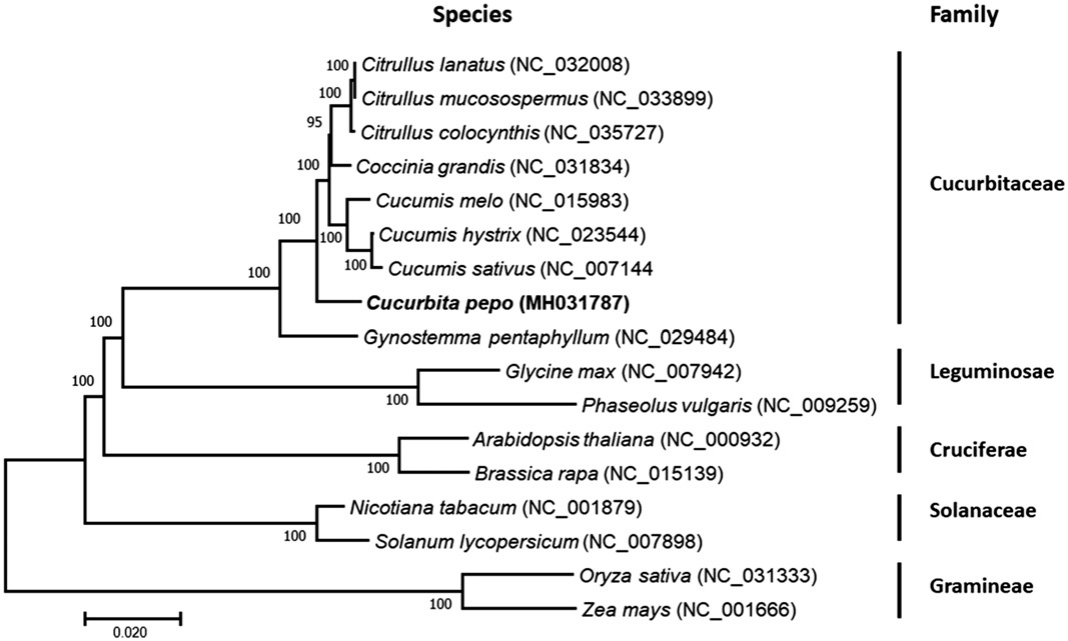
Phylogenetic tree showing relationship between *C. pepo* and other 16 species belonging to different families. Phylogenetic tree was constructed based on the complete chloroplast genomes using maximum likelihood (ML) with 1000 bootstrap replicates. Numbers in each the node indicated the bootstrap support values.
